# Socio-economic position and childhood multimorbidity: a study using linkage between the Avon Longitudinal study of parents and children and the general practice research database

**DOI:** 10.1186/1475-9276-12-66

**Published:** 2013-08-20

**Authors:** Rosie P Cornish, Andy Boyd, Tjeerd Van Staa, Chris Salisbury, John Macleod

**Affiliations:** 1 School of Social and Community Medicine, University of Bristol, Oakfield House, Oakfield Grove BS8 2BN, Bristol, UK; 2 Clinical Practice Research Datalink (CPRD), Medicines and Healthcare products Regulatory Agency (MHRA), London, UK

**Keywords:** Multimorbidity, Socio-economic position, Determinants, Childhood, Adolescence, Chronic conditions, ALSPAC, Linkage, GPRD

## Abstract

**Introduction:**

In adults, multimorbidity is associated with social position. Socially disadvantaged adults typically experience more chronic illness at a younger age than comparable individuals who are more advantaged. The relation between social position and multimorbidity amongst children and adolescents has not been as widely studied and is less clear.

**Methods:**

The NHS Information Centre (NHS IC) linked participants in the Avon Longitudinal Study of Parents and Children (ALSPAC) to the General Practice Research Database (GPRD). Multimorbidity was measured in three different ways: using a count of the number of drugs prescribed, a count of chronic diseases, and a person’s predicted resource use score; the latter two measures were derived using the Johns Hopkins ACG system. A number of different socio-economic position variables measured as part of ALSPAC during pregnancy and early childhood were considered. Ordered logistic and negative binomial regression models were used to investigate associations between socio-economic variables and multimorbidity.

**Results:**

After mutually adjusting for the different markers of socio-economic position, there was evidence, albeit weak, that chronic condition counts among children aged from 0 to 9 years were higher among those whose mothers were less well educated (OR = 0.44; 95% confidence interval 0.18-1.10; p = 0.08). Conversely, children whose mothers were better educated had higher rates of chronic illness between 10 and 18 years (OR = 1.94; 95% CI 1.14-3.30). However, living in a more deprived area, as indicated by the Townsend score, was associated with a higher odds of chronic illness between 10 and 18 years (OR for each increasing decile of Townsend score = 1.09; 95% CI 1.00-1.19; p = 0.06).

**Conclusions:**

We have found some evidence that, in younger children, multimorbidity may be higher amongst children whose parents are less well educated. In older children and adolescents this association is less clear. We have also demonstrated that linkage between prospective observational studies and electronic patient records can provide an effective way of obtaining objectively measured outcome variables.

## Introduction

Multimorbidity, the co-occurrence of two or more chronic medical conditions, is increasingly common [1,2] and has a large impact on a number of different outcomes, including treatment complications, disability, mortality and quality of life [3,4]. People with multimorbidity also account for a large proportion of healthcare usage, not only because they have multiple conditions, but also due to their relatively complex needs [2,4]. Although multimorbidity increases dramatically with age, one study found that the ratio of observed to expected frequency (expected figures based on the assumption that individual diseases occur independently) of subjects with three or more different chronic conditions was actually highest among those aged 0 to 19 years [5]. Estimates of the prevalence of multimorbidity vary quite substantially, depending on the definition and number of chronic conditions included - among those aged under twenty the estimates vary from under five percent [1,6] to around ten percent [5].

The fact that socio-economic position has an impact on health has been known for a long time. In 1980 the Black report was published, showing that there were large differences in both mortality and morbidity by social class in the UK [7]. In 2010, the Marmot review concluded that these inequalities still exist and that they accumulate across the lifecourse, starting prenatally [8]. Few studies have examined the impact of socio-economic position in childhood on multimorbidity. One study carried out in the USA examined the impact of childhood economic hardship on multimorbidity among people aged 50 or over and found that, on average, those who experienced hardship during childhood had a greater number of chronic conditions [9]. Others have found a relationship between multimorbidity and educational level [4,5,10] but only one of these studies [5] included children in their study. A recent study, carried out in Scotland, found that people living in more deprived areas were more likely to be multimorbid; this applied at all ages except among those aged 85 years or older [1]. Other studies have shown social class inequalities in rates of common childhood conditions, such as injury, asthma, respiratory infections, and general morbidity [11,12].

It has been suggested that social disadvantage increases the likelihood of childhood illness through increased exposure to conditions that are detrimental to health, such as poor diet, poor housing conditions, exposure to smoking, and psychosocial stress [13].

In this study, carried out as part of a larger project being conducted to explore the potential value of linkage between large observational cohort studies and administrative and healthcare data, we have investigated whether there is an association between socio-economic position measured during pregnancy and multimorbidity during childhood and adolescence.

## Methods

### Subjects

Subjects were those eligible to participate in ALSPAC who also had a record in the GPRD, which has now become part of the Clinical Practice Research Datalink [14]. The GPRD is an anonymised database of primary care records of around 5 million patients in the UK; practices using Vision software (this replaced VAMP Medical software) contribute to the database. Patients are part of the GPRD from the time at which they register into a practice that contributes to the database; similarly they leave the GPRD if they transfer to a practice that does not contribute. ALSPAC has been described in detail before [15]. To summarise, 20248 pregnant women living in and around Bristol, UK with due dates between 1 April 1991 and 31 December 1992 were eligible to take part in the study; of these, 14541 were recruited in 1990–1992 and a further 706 in later years. Among these, there were a total of 14775 live births. ALSPAC participants have been followed up regularly since birth. (Please note that the ALSPAC website contains details of all the data that is available through a fully searchable data dictionary [16]). Ethical approval was obtained from the ALSPAC Ethics and Law Committee, Local Research Ethics Committees and the NHS National Information Governance Board (NIGB).

### Linkage between ALSPAC and the GPRD

Linkage between ALSPAC and the GPRD was conducted by the NHS Information Centre (NHS IC) in the role of a trusted third party and using a methodology to preserve anonymity. The NHS numbers of individuals meeting recruitment criteria and eligible to participate in ALSPAC were ascertained by the NHS IC as part of a previous linkage exercise [15]. With approval from the NIGB Ethics and Confidentiality Committee, the NHS IC used this information to identify ALSPAC eligible individuals who also appeared in the GPRD; they then sent an anonymised linking dataset to be stored securely at the GPRD. ALSPAC and GPRD data for linked individuals were merged and analysed in a safe setting at the GPRD offices. As GPRD is anonymous and collected on an opt-out basis, and anonymity was preserved using the safeguards described above, this piece of research does not require consent above and beyond the consent obtained for participation in ALSPAC. However, ALSPAC has been collecting consent from participants, who are now adults, for ongoing participation in the study as well as consent to extract information from health and other administrative records and any participants who withdrew from the study or did not agree to their health records being extracted were excluded from the linkage.

### Social position data

A number of different measures of social position (collected by ALSPAC) were used. The following variables collected via postal questionnaires filled in by the mothers during pregnancy were included in the analysis: mother’s and father’s educational level; mother’s and father’s occupational social class; housing tenure; and family adversity index, a composite measure of social adversity, taking into account a variety of different risk factors thought to be important for mental health outcomes. These risk factors include items relating to housing adequacy, financial difficulties, family size and problems, maternal age and educational level, the availability of social and financial support, partner relationship, substance abuse, and crime [17]. Each subject’s Townsend score, based on their address at study enrolment and converted to deciles, was also included in the analysis. Social class was defined using the UK Registrar General’s occupational coding (SOC 90); family occupational social class was defined as the lower of maternal or paternal social class.

### Multimorbidity measures

Multimorbidity was measured in three different ways. The Johns Hopkins University Adjusted Clinical Groups (ACG®) System [18] was used to construct two of the measures. The first measure considered all diagnoses recorded up until the time at which an individual exited from the GPRD. The ACG system considers all the relevant Read codes from a person’s record and categorises these as one of 267 expanded diagnostic clusters (EDCs), a classification of clinically similar conditions. Salisbury et al. [2] previously defined 114 of these EDCs as chronic conditions; with the addition of the following EDCs in version 9.0 of the ACG software, this list has increased: EYE15 – age-related macular degeneration, PSY12 – bipolar disorder, and RHU05 – rheumatoid arthritis. We counted the number of chronic EDCs recorded for each person up to age 9 and between the ages of 10 and 18 years (inclusive). The second measure was the ACG itself, a mutually exclusive group based on a person’s combination of Aggregated Diagnostic Groups (ADGs). There are 32 ADGs; these are based on the expected subsequent resource use for a given condition and take into account its severity; whether it is acute, recurrent or chronic; the diagnostic certainty and aetiology of the condition; and whether or not it is likely to lead to specialist care. Every diagnosis code (in this case Read codes) is assigned to one of the 32 ADGs. If an individual has at least one diagnosis within a particular ADG then that person is classified as having that ADG. An individual’s ACG is based on their age, sex and the presence of particular ADGs, number of ADGs and number of major ADGs. The ACGs are assigned using diagnoses recorded over a one year period; we included each subject’s ACG whilst aged 5, 10, and 15 if they were registered in a GPRD practice for the entire year in question. If they were not in the GPRD for these particular time periods, we included each subject’s ACG while aged 4 or 6, 9 or 11, and 14 or 16, respectively; this was done to maximise the number of subjects available for analysis. Finally, and also as described by Brilleman and Salisbury [19], we counted the number of different drugs received by each subject while aged 0–9 years and while aged 10–18 years. As described in the above paper, each unique drug name was counted only once, and repeated prescriptions and prescriptions for different formulations or dosages of the same drug were not counted – this was done to try to reflect the number of unique conditions being treated during these two time periods.

### Statistical methods

#### Grouping of socio-economic position variables

To ensure adequate numbers in all categories of the socio-economic variables, some of the groups were combined: maternal and paternal education were analysed as O level or lower and A level or above; social classes I, II, and III non-manual were combined, as were III manual, IV and V; housing tenure was analysed as mortgaged/owned or other; and the family adversity index was regrouped as 0, 1, 2, and 3 or more. Townsend score was put into quintiles for the cross-tabulations but analysed as a decile in the regression analyses.

#### Analyses of multimorbidity measures

The chronic condition count was analysed as an ordered categorical variable. Because there were relatively few individuals with more than two chronic conditions, the counts were regrouped as zero, one, and two or more chronic conditions. Cross-tabulations and ordinal logistic regression were used to investigate the relationship between chronic condition counts and socio-economic position. In ordinal logistic regression, the odds ratios are (in this case) interpreted as the relative odds of being multimorbid compared to having zero or one chronic condition or the relative odds of having one or more chronic conditions compared to having none (the model assumes these odds ratios are the same) for different levels of each socio-economic position variable. Because of the large number of ACGs (subjects in this study fell into a total of 20, 24 and 27 categories at ages 5, 10, and 15, respectively), these were grouped according to their Resource Utilisation Band (RUB), such that ACGs with similar expected subsequent resource use were combined into a single category. There are six possible RUBs: non-user, health user, low morbidity, moderate morbidity, high morbidity, and very high morbidity. Again, this was analysed as an ordered categorical variable. Drug counts were modelled using negative binomial regression.

#### Exclusion criteria

For all the outcome measures, analyses by socio-economic position were restricted to those with complete socio-economic position data and multiple regression models were used to mutually adjust for the different socio-economic variables. Additional restrictions based on completeness of GPRD follow-up were applied as follows. The analysis of drug counts was restricted to those with complete GPRD follow up for the relevant time periods (0–9 years or 10–18 years). This was done instead of analysing the drug counts as rates because, assuming that many children receive some drugs, antibiotics for example, several times during their childhood for different infections, in the latter analysis the contribution of drugs for acute conditions to the overall drug count would be proportionately higher for those followed for a short amount of time compared to those followed for a longer period. The analysis of chronic condition counts between 0 and 9 years was restricted to those who registered into a GPRD practice before the age of five; this is explained in greater detail below. All analyses were carried out using Stata v12.

## Results

### Socio-economic position variables

**Figure 1:**
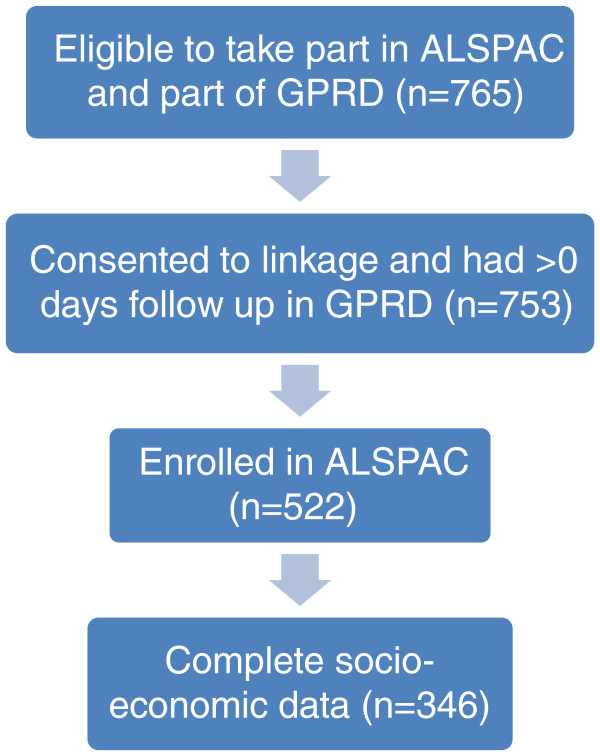
Flowchart showing numbers of subjects linked and included.

Of all the live births linked by the NHS IC, 765 appeared in the GPRD; eleven of these declined consent for linkage to their health records and one registered into and left GPRD on the same day, resulting in no follow up. Of the remaining 753 eligible individuals, 522 (69%) had enrolled in ALSPAC. Of these, 50% were female. As described above, apart from the Townsend score, which was available for all whom a record of contact was made during 1990–92, the socio-economic variables were only available for individuals who had enrolled in ALSPAC; in addition, numbers with a non-missing or valid response varied according to when the information was collected. Table 1 shows the distributions of each of the socio-economic position variables as well as the numbers with data available for each. Of the 522 patients contributing to ALSPAC and GPRD, 346 patients had complete data on all the socio-economic variables used in this study. These numbers are shown in Figure 1.

**Table 1:** Distribution of socio-economic position markers

**Socio-economic marker**		**Frequency (%)**
Maternal education (n = 460)	CSE/vocational/lower	153 (33.3%)
	O level	182 (39.6%)
	A level	85 (18.5%)
	Degree	40 (8.7%)
Paternal education (n = 376)	CSE/vocational/lower	99 (26.3%)
	O level	90 (23.9%)
	A level	127 (33.8%)
	Degree	60 (16.0%)
Occupational social class (n = 419)	I	13 (3.1%)
	II	91 (21.7%)
	III non-manual	93 (22.2%)
	III manual	118 (28.2%)
	IV	84 (20.1%)
	V	20 (4.8%)
Housing tenure during pregnancy (n = 483)	Mortgaged/owned	320 (66.3%)
	Private rented	33 (6.8%)
	Council rented/housing association	130 (26.9%)
Long family adversity index during pregnancy (n = 484)	0	169 (34.9%)
	1	133 (27.5%)
	2	81 (16.7%)
	3	42 (8.7%)
	4	17 (3.5%)
	5	15 (3.1%)
	6	12 (2.5%)
	7	11 (2.3%)
	8 or 9	4 (0.8%)
Townsend score (n = 620)	1^st^ decile (least deprived)	63 (10.2%)
	2	76 (12.3%)
	3	40 (6.5%)
	4	57 (9.2%)
	5	69 (11.1%)
	6	68 (11.0%)
	7	59 (9.5%)
	8	86 (13.9%)
	9	46 (7.4%)
	10^th^ (most deprived)	56 (9.0%)

### Multimorbidity measures

**Figure 2:**
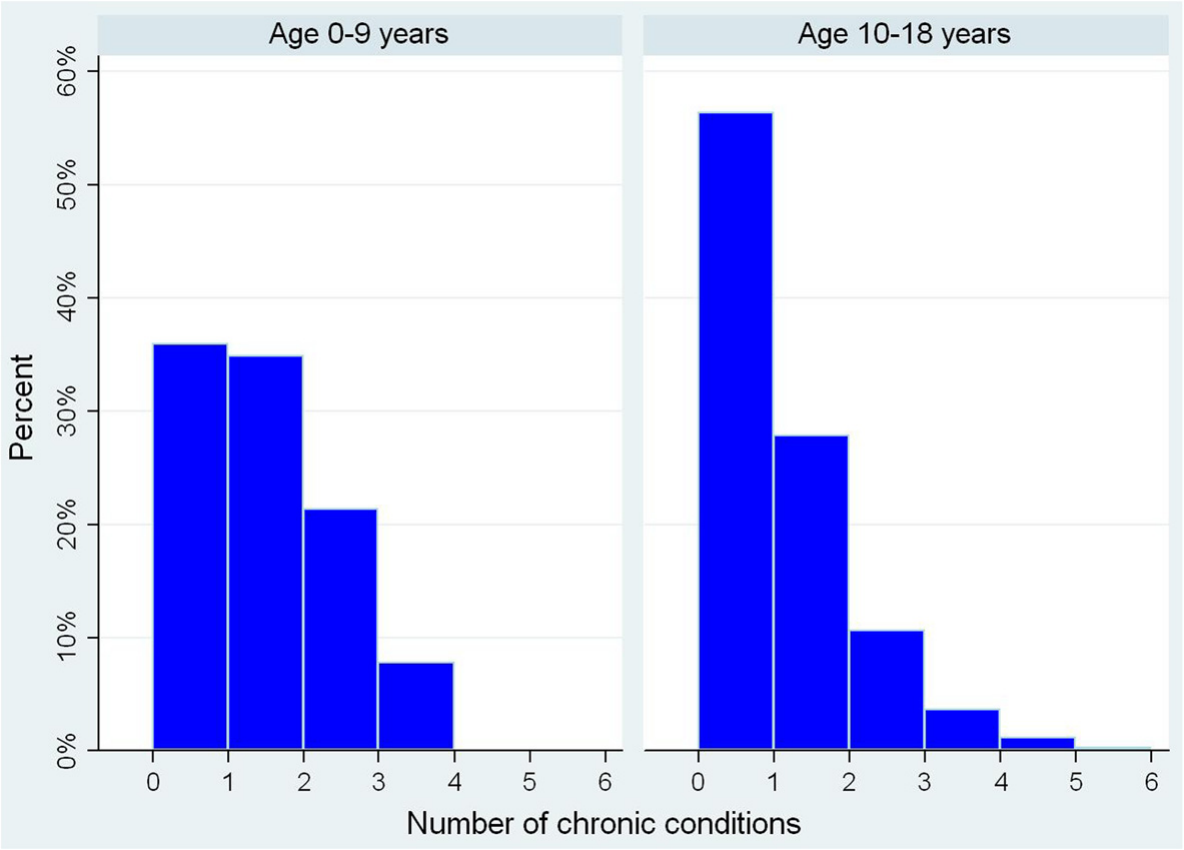
Distribution of chronic condition counts (0–9 and 10–18 years).

Between 0 and 9 years, 418 (56%) of the 753 children had no chronic conditions diagnosed. Among the 335 children with at least one chronic condition, there were a total of 517 chronic condition diagnoses. By far the most common conditions between these ages were dermatitis and eczema (192 children) and asthma (141 children), followed by deafness/hearing loss (24 children), seizure disorder (23 children) and anxiety/neuroses (20 children). Of the 662 children registered in a GPRD practice between the ages of 10 and 18, 373 (56%) were diagnosed with at least one chronic condition. A total of 442 chronic conditions were diagnosed during this time period. Again, the most common conditions were dermatitis and eczema (95 children) and asthma (92 children), followed by anxiety/neuroses (47 children). When the analysis was restricted to those who had registered into a practice contributing to the GPRD before the age of five, the percentage with no chronic conditions recorded between the ages of 0 and 9 years fell from 56% to 36%. Thus, further analysis on chronic condition counts for those aged 0 to 9 was restricted to this group in order to make the children included similar with respect to completeness of follow-up. Conversely, restricting to those with complete follow up between 10 and 18 years did not change the percentages markedly. Additionally restricting to those with socio-economic data from ALSPAC had very little impact on the percentages at either ages. Figure 2 shows the distribution of chronic condition counts between 0–9 years (for the restricted group) and 10–18 years. Among the 82 children registered before the age of five and with two or more chronic conditions between 0 and 9 years, 39 (47.6%) had both asthma and eczema. Similarly, among the 104 children with two or more chronic conditions between 10 and 18 years, 24 (23.1%) had both asthma and eczema.

**Figure 3:**
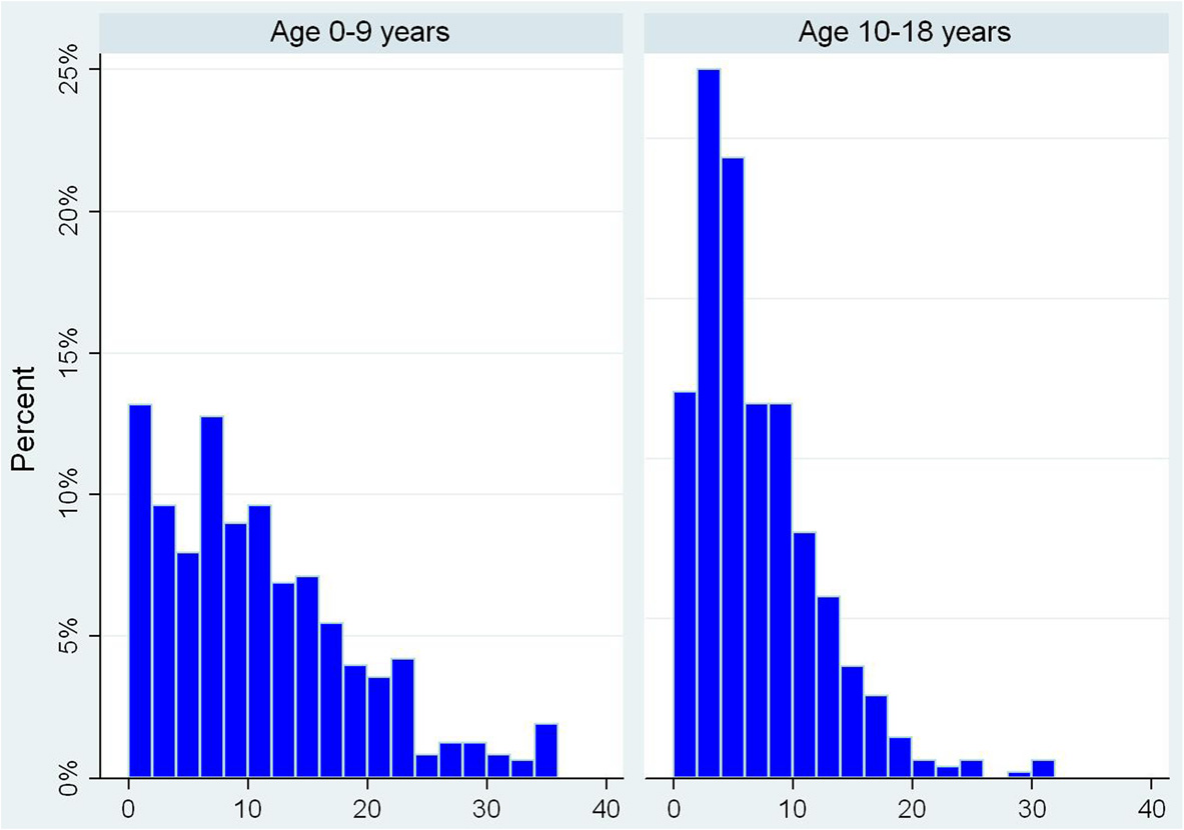
Distribution of drug counts (0–9 and 10–18 years).

Drug counts while aged 0 to 9 years and 10 to18 years are shown in Figure 3. The proportions with no prescribed drugs at these ages were 7% and 0%, respectively; the median (IQR) number of unique drugs prescribed were 9 (4–15) and 5 (3–9).

The most common ACGs (those individually accounting for at least 4% of subjects at any one of the three ages considered) at each age considered in this analysis are shown in Table 2. Apart from “non-user” (children with no GP consultations) and “no diagnoses”, the most common ACG at each age was acute minor, accounting for 20.6% of subjects at age 5, 16.2% at age 10 and 15.4% at age 15 years. The proportion with no diagnoses increased from 6.3% at 5 years to 22.8% at 15; there was a corresponding decrease in the proportion in the “acute minor and likely to recur” category (19.3% at 5 years and 5.3% at 15). The proportion of children with no consultations (“non-users”) also varied with age, being highest at 10 years and lowest at 15. Table 2 also shows the ACGs grouped according to their Resource Utilisation Band.

**Table 2:** ACGs at ages 5, 10 and 15

**ACG**	**Age 5**^**1**^	**Age 10**^**1**^	**Age 15**^**1**^
Non-user	68 (22.6%)	119 (28.7%)	78 (17.9%)
No diagnoses	19 (6.3%)	77 (18.6%)	99 (22.8%)
Acute minor	62 (20.6%)	67 (16.2%)	67 (15.4%)
Acute minor and likely to recur (with or without allergies)	58 (19.3%)	26 (6.3%)	23 (5.3%)
Likely to recur (with or without allergies)	29 (9.6%)	38 (9.2%)	46 (10.6%)
Acute minor and major	13 (4.3%)	18 (4.4%)	9 (2.1%)
Acute major	6 (2.0%)	11 (2.7%)	24 (5.5%)
2–3 other ADGs	16 (5.3%)	16 (3.9%)	29 (6.7%)
Other ACGs	30 (10.0%)	42 (10.1%)	60 (13.8%)
	301	414	435
Resource Utilisation Band			
Non-user	68 (22.6%)	119 (28.7%)	78 (17.9%)
Healthy user^2^	87 (28.9%)	150 (36.2%)	177 (40.7%)
Low morbidity^2^	124 (41.2%)	131 (31.6%)	151 (34.7%)
Moderate morbidity^2^	22 (7.3%)	14 (3.4%)	28 (6.4%)
High morbidity^2^	0	0	1 (0.2%)
Very high morbidity^2^	0	0	0
	301	414	435

^1^ Or at ages 4 or 6, 9 or 11, and 14 or 16, if data not available at ages 5, 10 and 15, respectively.

^2^ ACGs occurring in these data that fall into these categories are as follows:

Healthy user: no diagnoses, acute minor, eye and dental, preventive/administrative.

Low morbidity: acute major, likely to recur (with or without allergies), asthma, chronic medical (stable), chronic specialty (stable or unstable), psychosocial (stable), acute minor & major, acute minor & likely to recur (with or without eye & dental in addition to these), acute minor and chronic medical (stable), acute minor & psychosocial (stable), 2–3 other ADG combinations.

Moderate morbidity: Acute major & likely to recur, acute minor & likely to recur & psychosocial, psychosocial (unstable), acute minor & major & likely to recur (with or without stable chronic medical or psychosocial in addition to these), acute minor & psychosocial (unstable), 4-5/6-9 other ADGs (none major).

High morbidity: pregnancy plus 2–3 ADGs (none major), delivered.

Very high morbidity: N/A – none occurred in these data.

### Relationship between multimorbidity measures and socio-economic position

#### Chronic condition counts

Cross-tabulations of chronic condition counts against socio-economic position variables are given in Table 3. In the univariate analysis chronic condition counts between 0 and 9 years were related to maternal education. Children whose mothers had a lower level of education were more likely to have chronic conditions, particularly two or more chronic conditions. The relationship between maternal education and chronic condition counts at ages 10–18 years was reversed: those whose mothers had a lower educational level were less likely to have two or more chronic conditions. In the multivariate analysis, mutually adjusting for the different markers of socio-economic position, the relationship between chronic conditions between 0 and 9 years and maternal education became slightly weaker. The odds ratio (OR) for A level or degree versus O level or lower was 0.44 (95% confidence interval (CI) 0.18-1.10; p = 0.08). Between 10 and 18 years maternal education remained associated with chronic conditions, (OR for A level or degree versus O level or lower = 1.94; 1.14-3.30; p = 0.02). In addition, after adjustment there was some evidence of an association with housing tenure (OR for rented versus owned/mortgaged = 0.56; 0.31-1.02; p = 0.06) and Townsend score (OR for increasing deprivation decile = 1.09; 1.00-1.19; p = 0.06). When restricting to those with complete follow up while aged 0–9, the relationship between chronic conditions and maternal education remained more or less the same (OR = 0.43; 0.11-1.60), although the confidence interval became wider as a result of the reduced sample size. In contrast, the relationship with maternal education at ages 10–18 disappeared (OR = 0.95; 0.44 -2.06). However, the relationship with housing tenure became stronger (OR = 0.39; 0.17-0.89) and the relationship with Townsend score remained the same (OR =1.09; 0.96-1.24).

**Table 3:** Univariate analysis (unadjusted results) of chronic conditions by socio-economic position variables

**Socio-economic marker**		**Number of chronic conditions while aged 0–9 years (% within SEP category)**			**χ**^**2**^**; p-value**
		0	1	2 or more	
Mother’s education	O level or lower	35 (34.3%)	34 (33.3%)	33 (32.4%)	χ_2_^2^ = 7.9; p = 0.02
	A level or degree	20 (58.8%)	10 (27.4%)	4 (11.8%)	
Father’s education	O level or lower	23 (31.5%)	27 (37.0%)	23 (31.5%)	χ_2_^2^ = 4.3; p = 0.1
	A level or degree	32 (50.8%)	17 (27.0%)	14 (22.2%)	
Family social class	I, II, III non-manual	31 (49.2%)	18 (28.6%)	14 (22.2%)	χ_2_^2^ = 3.8; p = 0.1
	III manual, IV V	24 (32.9%)	26 (35.6%)	23 (31.5%)	
Housing tenure (in pregnancy)	Owned/mortgaged	48 (42.9%)	35 (31.3%)	29 (25.9%)	χ_2_^2^ = 1.0; p = 0.5
	Other	7 (29.2%)	9 (37.5%)	8 (33.3%)	
Family adversity index	0	25 (44.6%)	18 (32.1%)	13 (23.2%)	χ_6_^2^ = 4.4; p = 0.6
	1	17 (42.5%)	13 (32.5%)	10 (25.0%)	
	2	6 (30.0%)	5 (25.0%)	9 (45.0%)	
	≥3	7 (35.0%)	8 (40.0%)	5 (25.0%)	
Townsend score (quintile)	1st (least deprived)	18 (40.9%)	15 (34.1%)	11 (25.0%)	χ_6_^2^ = 2.1; p = 0.9
	2	11 (40.7%)	7 (25.9%)	9 (33.3%)	
	3	8 (33.3%)	10 (41.7%)	6 (25.0%)	
	4^th^ & 5^th^ (most deprived)^1^	18 (43.9%)	12 (29.3%)	11 (26.8%)	
		Number of chronic conditions while aged 10–18 years (% within SEP category)			
		0	1	2 or more	
Mother’s education	O level or lower	121 (58.7%)	60 (29.1%)	25 (12.1%)	χ_2_^2^ = 9.2; p = 0.01
	A level or degree	45 (45.5%)	29 (29.3%)	25 (25.3%)	
Father’s education	O level or lower	85 (54.1%)	49 (31.2%)	23 (14.7%)	χ_2_^2^ = 1.1; p = 0.6
	A level or degree	81 (54.7%)	40 (27.0%)	27 (18.2%)	
Family social class	I, II, III non-manual	79 (49.7%)	48 (30.2%)	32 (20.1%)	χ_2_^2^ = 4.3; p = 0.1
	III manual, IV V	87 (59.6%)	41 (28.1%)	18 (12.3%)	
Housing tenure	Mortgaged/owned	118 (51.5%)	70 (30.6%)	41 (17.9%)	χ_2_^2^ = 3.3; p = 0.2
	Other	48 (63.2%)	19 (25.0%)	9 (11.8%)	
Family adversity index	0	60 (49.6%)	43 (35.5%)	18 (14.9%)	χ_6_^2^ = 4.5; p = 0.6
	1	53 (56.4%)	25 (26.6%)	16 (17.0%)	
	2	29 (61.7%)	10 (21.3%)	8 (17.0%)	
	≥3	24 (55.8%)	11 (25.6%)	8 (18.6%)	
Townsend score (quintile)	1^st^ (least deprived)	51 (58.0%)	25 (28.4%)	12 (13.6%)	χ_8_^2^ = 4.1; p = 0.8
	2	31 (55.4%)	16 (28.6%)	9 (16.1%)	
	3	35 (48.0%)	21 (28.8%)	17 (23.3%)	
	4	33 (55.0%)	18 (30.0%)	9 (15.0%)	
	5^th^ (most deprived)	16 (57.1%)	9 (32.1%)	3 (10.7%)	

1. Groups combined as numbers small.

#### Drug counts

Maternal education was not related to drug counts at either age group: the rate ratio (RR) for A level or higher compared to O level or lower was 0.87 (0.64-1.18; p = 0.4) at ages 0–9 years and 1.01 (0.79-1.28; p = 1.0) at ages 10–18 years. The only socio-economic position variable associated with drug counts between 0 and 9 years was paternal education: RR for A level or higher compared to O level or lower = 0.78 (0.62-0.99; p = 0.04). None of the socio-economic position variables was associated with drug counts between 10 and 18 years. These results, together with equivalent results for the other socio-economic position variables are shown in Table 4. These results remained the same after mutual adjustment (adjusted RRs not shown).

**Table 4:** Univariate analysis (unadjusted results) of drug counts by socio-economic position variables

**Socio-economic marker**		**Drug counts age 0-9**		
		Number of drugs/person	Rate ratio (95% CI)	p-value
Mother’s education	O level or lower	1107/69 = 16.0	1.00	p = 0.4
	A level or degree	195/14 = 13.9	0.87 (0.64, 1.18)	
Father’s education	O level or lower	902/53 = 17.0	1.00	p = 0.04
	A level or degree	400/30 = 13.3	0.78 (0.62, 0.99)	
Family social class	I, II, III non-manual	506/33 = 15.3	1.00	p = 0.8
	III manual, IV V	796/50 = 15.9	1.04 (0.82, 1.32)	
Housing tenure (in pregnancy)	Owned/mortgaged	1096/69 = 15.9	1.00	p = 0.3
	Other	206/14 = 14.7	0.93 (0.68, 1.26)	
Family adversity index	0	498/31 = 16.1	1.00	p = 0.6
	1	404/25 = 16.2	} 0.97 (0.88, 1.09)	
	2	199/14 = 14.2		
	≥3	201/13 = 15.5		
Townsend score (quintile)	1^st^ (least deprived)	404/25 = 16.2	1.00	p = 0.9
	2	250/18 = 13.9	} 1.00 (0.95, 1.04)^2^	
	3	309/18 = 17.2		
	4 & 5^th^ (most deprived)^1^	339/22 = 15.4		
		Drug counts age 10-18		
		Number of drugs/person	Rate ratio	p-value
Mother’s education	O level or lower	758/95 = 8.0	1.00	p = 1.0
	A level or degree	410/51 = 8.0	1.01 (0.79, 1.28)	
Father’s education	O level or lower	634/75 = 8.5	1.00	p = 0.3
	A level or degree	534/71 = 7.5	0.89 (0.71, 1.12)	
Family social class	I, II, III non-manual	601/80 = 7.5	1.00	p = 0.3
	III manual, IV V	567/66 = 8.6	1.14 (0.91, 1.44)	
Housing tenure	Mortgaged/owned	922/113 =8.2	1.00	p = 0.5
	Other	246/33 = 7.5	0.91 (0.69, 1.21)	
Family adversity index	0	452/63 = 7.2	1.00	p = 0.2
	1	430/49 = 8.8	} 1.07 (0.96, 1.21)	
	2	151/19 = 7.9		
	≥3	135/15 = 9.0		
Townsend score (quintile)	1^st^ (least deprived)	414/54 = 7.7	1.00	p = 0.6
	2	152/23 = 6.6	} 1.01 (0.97, 1.05)^2^	
	3	335/36 = 9.3		
	4	176/22 = 8.0		
	5^th^ (most deprived)	91/11 = 8.3		

1. Groups combined as numbers small.

2. Townsend score entered as a decile in the regression analysis.

#### Resource utilisation bands

Although there was no statistical evidence for a relationship between socio-economic position and predicted resource use at any of the ages considered, at 5 and 15 years the trends were in the same direction as for chronic condition counts, such that children with less well educated mothers and those from a lower occupational social class were more likely to be classified as having low or moderate morbidity (as opposed to being healthy or a non-user) at age 5 but less likely to be classified as such at age 15 years. These results are shown in Tables 5, 6 and 7.

**Table 5:** Univariate analysis (unadjusted results) of Resource Utilisation Bands at age 5 by socio-economic position variables

**Socio-economic marker**		**Resource Utilisation Band at age 5 (% within SEP category)**			
		**Non-user**	**Healthy user**	**Low or moderate morbidity**	**χ**^**2**^**; p-value**
Mother’s education	O level or lower	24 (22.0%)	32 (29.4%)	53 (48.6%)	χ_2_^2^ = 2.9; p = 0.2
	A level or degree	14 (35.9%)	9 (23.1%)	16 (41.0%)	
Father’s education	O level or lower	20 (26.0%)	23 (29.9%)	34 (44.2%)	χ_2_^2^ = 0.5; p = 0.8
	A level or degree	18 (25.4%)	18 (25.4%)	35 (49.3%)	
Family social class	I, II, III non-manual	23 (31.1%)	20 (27.0%)	31 (41.9%)	χ_2_^2^ = 2.4; p = 0.3
	III manual, IV V	15 (20.3%)	21 (28.4%)	38 (51.4%)	
Housing tenure (in pregnancy)	Owned/mortgaged	27 (22.5%)	38 (31.7%)	55 (45.8%)	χ_2_^2^ = 6.2; p = 0.05
	Other	11 (39.3%)	3 (10.7%)	14 (50.0%)	
Family adversity index	0	18 (28.1%)	19 (29.7%)	27 (42.2%)	χ_6_^2^ = 9.8; p = 0.1
	1	11 (26.2%)	6 (14.3%)	25 (59.5%)	
	2	4 (19.1%)	6 (28.6%)	11 (52.4%)	
	≥3	5 (23.8%)	10 (47.6%)	6 (28.6%)	
Townsend score (quintile)	1^st^ (least deprived)	9 (18.0%)	16 (32.0%)	25 (50.0%)	χ_6_^2^ = 4.2; p = 0.6
	2	11 (37.9%)	6 (20.7%)	12 (41.4%)	
	3	8 (28.6%)	8 (28.6%)	12 (42.9%)	
	4^th^ or 5^th^ (most deprived)^1^	10 (24.4%)	11 (26.8%)	20 (48.8%)	

1. Groups combined as numbers small.

**Table 6:** Univariate analysis (unadjusted results) of Resource Utilisation Bands at age 10 by socio-economic position variables

**Socio-economic marker**		**Resource Utilisation Band at age 10 (% within SEP category)**			
		**Non-user**	**Healthy user**	**Low or moderate morbidity**	**χ**^**2**^**; p-value**
Mother’s education	O level or lower	45 (35.4%)	37 (29.1%)	45 (35.4%)	χ_2_^2^ = 2.3; p = 0.3
	A level or degree	20 (29.4%)	27 (39.7%)	21 (30.9%)	
Father’s education	O level or lower	32 (32.7%)	30 (30.6%)	36 (36.7%)	χ_2_^2^ = 0.8; p = 0.7
	A level or degree	33 (34.0%)	34 (35.1%)	30 (30.9%)	
Family social class	I, II, III non-manual	35 (34.3%)	31 (30.4%)	36 (35.3%)	χ_2_^2^ = 0.6; p = 0.7
	III manual, IV V	30 (32.3%)	33 (35.5%)	30 (32.3%)	
Housing tenure (in pregnancy)	Owned/mortgaged	47 (31.3%)	54 (36.0%)	49 (32.7%)	χ_2_^2^ = 3.0; p = 0.2
	Other	18 (40.0%)	10 (22.2%)	17 (37.8%)	
Family adversity index	0	31 (36.9%)	27 (32.1%)	26 (31.0%)	χ_6_^2^ = 3.1; p = 0.8
	1	17 (29.3%)	19 (32.8%)	22 (37.9%)	
	2	12 (40.0%)	9 (30.0%)	9 (30.0%)	
	≥3	5 (21.7%)	9 (39.1%)	9 (39.1%)	
Townsend score (quintile)	1^st^ (least deprived)	19 (28.8%)	24 (36.4%)	23 (34.9%)	χ_6_^2^ = 13.5; p = 0.04
	2	10 (28.6%)	17 (48.6%)	8 (22.9%)	
	3	17 (38.6%)	6 (13.6%)	21 (47.7%)	
	4^th^ and 5^th^ (most deprived)^1^	19 (38.0%)	17 (34.0%)	14 (28.0%)	

1. Groups combined as numbers small.

**Table 7:** **Univariate analysis** (**unadjusted results) of Resource Utilisation Bands at age 15 by socio-economic position variables**

**Socio-economic marker**		**Resource Utilisation Band at age 15 (% within SEP category)**			
		**Non-user**	**Healthy user**	**Low or moderate morbidity**	**χ**^**2**^**; p-value**
Mother’s education	O level or lower	28 (20.3%)	56 (40.6%)	54 (39.1%)	χ_2_^2^ = 5.4; p = 0.07
	A level or degree	7 (9.3%)	29 (38.7%)	39 (52.0%)	
Father’s education	O level or lower	17 (15.6%)	47 (43.1%)	45 (41.3%)	χ_2_^2^ = 1.0; p = 0.6
	A level or degree	18 (17.3%)	38 (36.5%)	48 (46.2%)	
Family social class	I, II, III non-manual	16 (13.6%)	46 (39.0%)	56 (47.5%)	χ_2_^2^ = 2.3; p = 0.3
	III manual, IV V	19 (20.0%)	39 (41.1%)	37 (39.0%)	
Housing tenure (in pregnancy)	Owned/mortgaged	27 (16.5%)	67 (40.9%)	70 (42.7%)	χ_2_^2^ = 0.3; p = 0.9
	Other	8 (16.3%)	18 (36.7%)	23 (46.9%)	
Family adversity index	0	16 (18.8%)	32 (37.7%)	37 (43.5%)	χ_6_^2^ = 3.2; p = 0.8
	1	10 (14.9%)	27 (40.3%)	30 (44.8%)	
	2	7 (18.9%)	17 (46.0%)	13 (35.1%)	
	≥3	2 (8.3%)	9 (37.5%)	13 (54.2%)	
Townsend score (quintile)	1^st^ (least deprived)	10 (15.4%)	26 (40.0%)	29 (44.6%)	χ_6_^2^ = 2.8; p = 0.8
	2	9 (21.4%)	19 (45.2%)	14 (33.3%)	
	3	8 (16.7%)	17 (35.4%)	23 (47.9%)	
	4^th^ and 5^th^ (most deprived)^1^	8 (13.8%)	23 (39.7%)	27 (46.6%)	

Groups combined as numbers small.

## Discussion

We have shown that multimorbidity is quite common among children - in this linked dataset 16% of 10–18 year olds had two or more chronic conditions and the corresponding figure among 0–9 year olds could be as high as 29%. This figure is much higher than figures from other studies [1,5,6]. This is likely to be mainly due to the differing definitions and numbers of chronic conditions included. For example, in the Scottish study, 40 chronic conditions were included and, in the case of asthma and eczema, the most common conditions occurring among children in our study, they had to have received treatment for these conditions in the past 12 months for them to be counted [5]. We have included more than one hundred chronic conditions and have counted them if they occurred during a much longer time period. Our estimates of multimorbidity are thus likely to be less conservative.

Although our conclusions should be regarded as tentative, we have also provided some evidence that multimorbidity may be associated with socio-economic position: in this study, higher maternal education was associated with lower levels of multimorbidity among those aged 0 to 9 years; in contrast, among those aged 10 to 18 years, higher socio-economic position, as measured by housing tenure and maternal education, was associated with higher levels of multimorbidity, although the latter relationship disappeared in some analyses. In contrast, when these factors were taken into account, increasing neighbourhood deprivation was associated with increasing multimorbidity in this older age group. Similar results have been found by others. Chen and colleagues carried out a review of studies looking at the relationship between socio-economic position and child health. One of their main objectives was to examine whether this relationship changed with age. They found evidence that lower socio-economic position was associated with a higher prevalence of asthma at younger ages (up to age nine), but either no relationship or a reverse relationship during adolescence, although the relationship with severe asthma appeared to persist throughout childhood [12]. They hypothesised that this could be because certain characteristics associated with low socio-economic position, such as poor housing conditions, which, in turn, are related to asthma, could become less important during adolescence when individuals spend relatively more time outside the home environment. Further, they suggested that neighbourhood characteristics are likely to play a more important role in adolescent health. Similarly, another review concluded that there is a relative equalisation during adolescence in terms of rates of chronic and acute illness, certain dimensions of mental health, and several other outcomes [20]. There are conflicting results in the literature regarding eczema, the other main chronic condition occurring among the children in this study. One study found that the prevalence of eczema was higher among higher socioeconomic groups [21], whereas others have found no relationship [22]. The fact that there may be different, even opposing, effects of socio-economic position on different chronic illnesses occurring among children may result in there being only a weak association between multimorbidity and socio-economic position. A large proportion of the children with chronic illnesses had either asthma or eczema or both. Therefore, it should be acknowledged that the observed relationships between socio-economic position and multimorbidity in this study are mainly, although not entirely, a reflection of an observed association with atopic illnesses.

### Strengths and limitations

Our study has two main strengths. Firstly, we were able to look at a number of different measures of socio-economic position which were measured before the children were born, thus avoiding issues of reverse-causality. There may have been changes in some of the measures during the course of the study, particularly housing tenure and Townsend score; however, relative social position has been shown to be quite stable [23]. Furthermore, it is unlikely that there would have been substantial changes in parental educational levels or occupational social class; as such, it seems reasonable to assume that the values of these factors in pregnancy are fairly representative of childhood socio-economic background. The other main strength is that, by linking to the GPRD, we were able to obtain objectively-measured outcome variables, rather than relying on self-reported health.

One of the limitations of this study is the fact that the children had differing periods of follow-up, as this was dependent on when they happened to be registered in a GP practice contributing to the GPRD. The median length of GPRD follow up was 8.2 years, but this ranged from just over five weeks to the full nineteen years under consideration. Since GPRD contains a representative sample of the UK population and subjects enter and exit the GPRD when they register with or leave a GP practice that contributes to this database, it is unlikely that the relationship between socio-economic position and multimorbidity would be different among those with complete and incomplete follow up. However, although historic diagnoses are contained within a person’s electronic patient record – and this is likely to be particularly the case for chronic conditions – we cannot be certain that they are complete. When the analysis was restricted to those who entered the GPRD before the age of five, the percentage with at least one chronic condition between 0 and 9 years changed quite dramatically. The most common chronic condition during this period was eczema. Atopic eczema is a common chronic condition, with onset typically in early childhood [24]. It has been shown that a relatively large proportion of eczema cases are mild and, in addition, atopic eczema often clears at some point during childhood [24]. As such, children who were not registered in a GPRD practice but had mild eczema during infancy may not have a diagnosis of eczema in their record. We tried to address this issue by restricting the main analysis to those who were registered in a GPRD practice before the age of five, thus making the sample more comparable in terms of their follow-up profile and our results indicate that the associations with socio-economic position were broadly similar when the analysis was carried out on a more restricted dataset. Because historic prescriptions are not added to a patient’s record when they join a practice we restricted the analysis of drug counts to those with complete GPRD for the periods in question; the main drawback of this is that the sample size decreased quite markedly, thus reducing the study’s power.

A second limitation relates to how we defined our outcome measures. In the study carried out by Salisbury and colleagues [2], the subjects were all adults. As discussed above, some of the chronic conditions included, particularly eczema, can be relatively mild and short-lived in children. However, by dividing childhood up into two distinct periods of follow-up, chronic conditions that were diagnosed in early childhood but had resolved before the age of ten would not be included as a chronic condition in later childhood, thereby addressing this issue to a certain extent. Obviously this would not take into account the fact that a diagnosis of dermatitis or eczema could refer to a very short-lived reaction to an irritant. When calculating the ACGs, we only used diagnoses recorded during a one-year period. In other words, previous diagnoses of chronic conditions that are still current but do not happen to be recorded during the year under consideration were not included in the calculation of a person’s ACG category. This may have affected the categorisation of some subjects. However, the findings at age 5 and 15 years were generally consistent with those for chronic conditions, suggesting that this did not have a substantial impact.

It should also be noted that the three different measures of multimorbidity used in this study assess slightly different aspects of morbidity. The count of chronic EDCs provides a measure of the total number of chronic conditions being experienced by a child in a particular time period (here from 0 to 9 and 10 to 18 years). The main drawback of this measure is that it takes no account of severity and duration; this is particularly an issue in this study because the most common chronic conditions were asthma and eczema which, among children, vary substantially with respect to these factors. The drug count includes drugs prescribed for both chronic and acute conditions. Although repeat acute conditions will only be counted once if the same drug is prescribed each time, a person with numerous short-lived conditions all treated with different drugs would be classified as having a greater level of multimorbidity using this measure than someone with one serious condition treated with only one or two different drugs. Finally, the ACGs and RUBs also consider acute conditions, although these measures, unlike the drug count, do take into account the usual severity of a condition and whether or not it is likely to recur. Thus, these measures assess a person’s overall morbidity burden as opposed to multimorbidity per se.

Although diagnosis data in the GPRD has been shown to be well recorded, particularly for chronic conditions [25], there are some drawbacks to using routine data to define outcome measures. Firstly, we are assuming that all diagnoses are recorded (and recorded correctly). Secondly, and perhaps more importantly for this particular study, having a diagnosis recorded by a GP depends on whether a child was actually taken to see the GP. Thus, higher rates of recorded illness could be genuinely due to higher morbidity but could also reflect higher parental anxiety, leading to higher consultation rates and hence a greater likelihood of being diagnosed and receiving prescriptions for certain conditions; conversely lower rates of recorded illness may not necessarily be due to lower morbidity - it could reflect neglect, for example, or perhaps inappropriate use of alternative services such as A&E. It is not possible to determine whether the observed associations between social position and multimorbidity were wholly a reflection of differing levels of morbidity or partly due to differences in help-seeking behaviour. Our measures of multimorbidity are only proxy measures and there is likely to be a certain amount of misclassification.

Finally, it should be added that the sample size in this study was quite small and a large number of statistical tests were carried out. Therefore, our conclusions can only be regarded as tentative. ALSPAC is currently in the process of trialling alternative methods of linking to GP data and we hope to be able to repeat these analyses on a larger sample in the future. This would also allow us to look in more detail at individual chronic conditions. With a larger dataset it would also be possible to try to determine the pathways through which social position might be impacting on multimorbidity.

In conclusion, we have found that in younger children apparent multimorbidity (as reflected in patient records from primary medical care services) appears to be higher amongst children whose mothers are less educated. In older children and adolescents this association is less clear. We have also demonstrated that linkage between prospective observational studies such as ALSPAC and electronic patient records can be an effective way of obtaining objectively measured outcome variables, including outcome measures for subjects who may have been lost due from the study.

## Competing interests

The authors declare that they have no competing interests.

## Authors’ contributions

RC carried out the statistical analysis and drafted the manuscript. AB established the linkage process and contributed to the drafting of the paper. TVS established the linkage and data management process and reviewed the manuscript. CS contributed to design of the study, interpretation of data and drafting of the paper. JM conceived the study and is PI of the Project to Enhance ALSPAC through Record Linkage (PEARL). All authors read and approved the final manuscript.

## References

[B1] Barnett K Mercer SW Norbury M Watt G Wyke S Guthrie B Epidemiology of multimorbidity and implications for health care, research, and medical education: a cross-sectional study Lancet 2012 380 9836 37 43 10.1016/S0140-6736(12)60240-2 22579043

[B2] Salisbury C Johnson L Purdy S Valderas JM Montgomery AA Epidemiology and impact of multimorbidity in primary care: a retrospective cohort study Br J Gen Pract 2011 61 582 e12 21 10.3399/bjgp11X548929 21401985 PMC3020068

[B3] Fortin M Soubhi H Hudon C Bayliss EA van den Akker M Multimorbidity's many challenges BMJ 2007 334 7602 1016 7 10.1136/bmj.39201.463819.2C 17510108 PMC1871747

[B4] Marengoni A Winblad B Karp A Fratiglioni L Prevalence of chronic diseases and multimorbidity among the elderly population in Sweden Am J Public Health 2008 98 7 1198 200 10.2105/AJPH.2007.121137 18511722 PMC2424077

[B5] van den Akker M Buntinx F Metsemakers JFM Roos S Knottnerus JA Multimorbidity in general practice: prevalence, incidence, and determinants of co-occurring chronic and recurrent diseases J Clin Epidemiol 1998 51 5 367 75 10.1016/S0895-4356(97)00306-5 9619963

[B6] Newacheck PW Stoddard JJ Prevalence and impact of multiple childhood chronic illnesses J Pediatr 1994 124 1 40 8 10.1016/S0022-3476(94)70252-7 7506774

[B7] Black D Morris J Smith C Townsend P Inequalities in health. Report of a research working group 1980 London: Department of Health and Social Security

[B8] Marmot M Fair society, healthy lives (The Marmot Review): strategic review of health inequalities in England post-2010 2010 London: University College London

[B9] Tucker-Seeley RD Li Y Sorensen G Subramanian SV Lifecourse socioeconomic circumstances and multimorbidity among older adults BMC Publ Health 2011 11 313 10.1186/1471-2458-11-313 PMC3118239 21569558

[B10] Nagel G Peter R Braig S Hermann S Rohrmann S Linseisen J The impact of education on risk factors and the occurrence of multimorbidity in the EPIC-Heidelberg cohort BMC Publ Health 2008 8 384 10.1186/1471-2458-8-384 PMC2614432 19014444

[B11] Weightman AL Addis SG Morgan HE Turley RL Mann MK Morgan FM Shepherd MA Social Determinants For Child Health: A Systematic Review 2008 Cardiff: Welsh Assembly Government

[B12] Chen E Matthews KA Boyce WT Socioeconomic differences in children's health: how and why do these relationships change with age? Psychol Bull 2002 128 2 295 329 11931521 10.1037/0033-2909.128.2.295

[B13] Shaw M Dorling D Gordon D Davey Smith G Explaining the gap. Chapter Three in The widening gap: Health inequalities and policy in Britain 1999 Bristol: The Policy Press

[B14] The Clinical Practice Research Datalinkhttp://www.cprd.com

[B15] Boyd A Golding J Macleod J Lawlor DA Fraser A Henderson J Molloy L Ness A Ring S Davey Smith G Cohort Profile: The ‘Children of the 90s’—the index offspring of the Avon Longitudinal Study of Parents and Children Int J Epidemiol 2012 42 1 111 127 http://dx.doi.org/10.1093/ije/dys064. Epub 2012 Apr 16 22507743 10.1093/ije/dys064 PMC3600618

[B16] The Avon Longitudinal Study of Parents and Childrenhttp://www.bris.ac.uk/alspac/researchers/data-access/data-dictionary

[B17] Steer C Wolke D ALSPAC Study Team An index of family adversity [poster], Presented at 2004 Bristol, United Kingdom: 3rd Conference on Epidemiological Longitudinal Studies in Europe

[B18] The Johns Hopkins University ACG systemhttp://acg.jhsph.org

[B19] Brilleman SL Salisbury C Comparing measures of multimorbidity to predict outcomes in primary care: a cross sectional study Family Practice 2013 30 2 172 178 http://dx.doi.org/10.1093/fampra/cms060. Epub 2012 Oct 8 10.1093/fampra/cms060 23045354 PMC3604888

[B20] West P Health inequalities in the early years: Is there equalisation in youth? Soc Sci Med 1997 44 6 833 858 10.1016/S0277-9536(96)00188-8 9080566

[B21] Williams HC Strachan DP Hay RJ Childhood eczema: disease of the advantaged? BMJ 1994 308 6937 1132 1135 10.1136/bmj.308.6937.1132 8173454 PMC2540131

[B22] Ruijsbroek A Smit HA Koppelman GH Kerkhof M Wijga AH Droomers M. The development of socio-economic health differences in childhood: results of the Dutch longitudinal PIAMA birth cohort. BMC Public Health 2011 11 225 10.1186/1471-2458-11-225 PMC3094243 21486447

[B23] Field F The Foundation Years: preventing poor children becoming poor adults. The report of the Independent Review on Poverty and Life Chances 2010 London: Cabinet Office

[B24] National Collaborating Centre for Women’s and Children’s Health Atopic Eczema in Children: management of atopic eczema in children from birth up to the age of 12 years. Clinical guideline commissioned by the National Institute for Health and Clinical Excellence (NICE) 2007 London: RCOG Press

[B25] Khan NF Harrison SE Rose PW Validity of diagnostic coding within the General Practice Research Database: a systematic review Br J Gen Pract 2010 60 572 e128 36 10.3399/bjgp10X483562 20202356 PMC2828861

